# Antimicrobial Symposium

**DOI:** 10.1155/S106474499500055X

**Published:** 1995

**Authors:** David E. Soper

**Affiliations:** Department of Obstetrics and Gynecology Medical University of South Carolina Division of Benign Gynecology 171 Ashley Avenue Charleston SC 29425-2233 USA


Infectious Diseases in Obstetrics and Gynecology 3:175 (1995)
© 1996 Wiley-Liss, Inc.

### Antimicrobial Symposium

The *Journal* recognizes the influx of new antimicrobials onto the clinical scene. Our desire is to offer the reader an up-to-date, succinct, and practical appraisal of these newer antimicrobials and the clinician a resource from which to make clinical decisions as to their use in practice. The *Journal* welcomes suggestions from its audience concerning other antimicrobials they would like to see reviewed.

This issue of *Infectious Diseases in Obstetrics and Gynecology* introduces a new section which will become a standing feature, "Antimicrobial Symposium." Anti-infective agents have been utilized for thousands of years. The discovery and clinical use of the sulfonamides in 1936 ushered in the modern era of chemotherapy. With the subsequent development of penicillin and streptomycin in the 1940s, the "golden age" of antimicrobial chemotherapy had arrived.[1] The continued development of effective antimicrobial drugs has revolutionized the management of infectious diseases over the past 50 years.

The pharmaceutical industry has long recognized the market for effective antimicrobials. With more than $2.4 billion being spent annually on oral antibiotics alone, these agents are some of the most commonly prescribed drugs dispensed by pharmacies in this country.[2] The continued development of agents with preferred pharmacokinetic profiles, oral dosing, and selective anti-infective spectra has improved the clinician's ability to treat infections by decreasing side effects and improving patient compliance. In addition, continued availability of anti-infectives in a timely manner to address emerging problems in infectious diseases and to overcome the difficulties posed by microbial resistance to older anti-infective drugs is of paramount importance.[3]

Infection remains the single most common clinical problem encountered by the obstetrician-gynecologist. An up-to-date understanding of the large number of new agents being introduced in the market is imperative. This section will focus on newer anti-infective agents in an attempt to allow the clinician to review the clinical applications of these agents with respect to their effectiveness, pharmacokinetics, side effects, and cost. We hope that this section will help you make a rational decision as to the utility of the reviewed agents in your practice. In addition, the compendium of articles at the year's end should serve as a valuable resource as you review your prescribing habits.

*David E. Soper*
*Department of Obstetrics and Gynecology*
*Medical University of South Carolina*
*Charleston, South Carolina*

### REFERENCES

1. Moellering RC: Principles of anti-infective therapy. In Mandell GL, Douglas RG, Bennett JE (eds): Principles and Practice of Infectious Diseases. New York: Churchill Livingstone, 1990.
2. Frieden TR, Mangi RJ: Inappropriate use of oral ciprofloxacin. JAMA 263:1438–1440, 1990.
3. Beam TR: Preface: IDSA. Clin Infect Dis 15(S):S1–S2, 1992.

Address correspondence/reprint requests to Dr. David E. Soper, Department of Obstetrics and Gynecology, Medical University of South Carolina, Division of Benign Gynecology, 171 Ashley Avenue, Charleston, SC 29425-2233.

Received November 28, 1995
Accepted November 30, 1995

Editorial
